# Studies of the genus *Enchodelus* Thorne, 1939 (Nematoda, Nordiidae) from Arctic polar deserts. 1. Species with long odontostyle: *E. makarovae* sp. n. and *E. groenlandicus* (Ditlevsen, 1927) Thorne, 1939, with an identification key to the species of the *E. macrodorus* group

**DOI:** 10.3897/zookeys.212.3464

**Published:** 2012-07-30

**Authors:** Milka Elshishka, Stela Lazarova, Vlada K. Peneva

**Affiliations:** 1 Institute of Biodiversity and Ecosystem Research, Bulgarian Academy of Sciences

**Keywords:** Taxonomy, morphology, morphometrics, Nematoda, cold desert, new geographic record

## Abstract

Two nematode species of the genus *Enchodelus* Thorne, 1939, one new and one known from Arctic polar deserts were studied. *Enchodelus makarovae* **sp. n.** is an amphimictic species, characterised by females with body length of 1.57–2.00 mm, lip region 15–17.5 µm wide, amphid duplex, odontostyle 38–43 µm long or 2.3–2.8 times lip region diam. Odontophore with flanges, 1.2–1.4 times as long as odontostyle; pharynx length 320–377 µm, pharyngeal expansion 113–130 µm long or 32–37% of total pharynx length; female genital system amphidelphic, uterus tripartite, *pars refringens vaginae* with two trapezoid sclerotisations, vulva a transverse slit (V=45–51%); tail bluntly conoid (25–35 µm, c=45.8–70.3, c’=0.6–0.9 in females, and 29–33 µm, c=46.4–58.9, c’=0.7–0.8 in males). Males with 65–74 µm long spicules and 10–12 spaced ventromedian supplements. Additional information for *Enchodelus groenlandicus* is provided, this being a new geographic record for the Putorana Plateau, Russian Arctic.

## Introduction

Currently, the genus *Enchodelus* Thorne, 1939 (Nordiidae, Pungentinae) contains 28 species distributed mainly in the northern hemisphere (Peña-Santiago et al. 2005); only one species (*Enchodelus signyensis* Loof, 1975) has been described from Antarctica. The members of the genus are common at high altitudes (1260-4400 m a.s.l) and latitudes, frequently associated with mosses and rock vegetation (Ahmad and Jairajpuri 1980, Eliava and Eliashvili 1990, Peneva et al. 2009). According to their feeding habits, representatives of the genus *Enchodelus* are attributed to the omnivorous trophic group (Yeates et al. 1993).

Ahmad and Jairajpuri (1980) provided a revision of the genus and grouped species into five subgenera(*Enchodelus*, *Paraenchodelus*, *Heterodorus*, *Rotundus*, *Nepalus*) on the basis of tail shape, odontostyle length, odontophore morphology and presence of a peculiar chamber in the female reproductive system. Recently, Guerrero et al. (2007a, 2008) divided the genus into three groups, based on tail shape and odontostyle length: species with long odontostyle (>35 μm) and rounded tail; species with medium size odontostyle (<35 μm) and rounded tail, and species with conical tail. The subgenera of *Enchodelus* are also not recognized by Andrássy (2009a) and species with conical tail are considered as belonging to the genus *Heterodorus* Altherr, 1952 (Andrássy 2011).

Here we provide data on two species of *Enchodelus* which belong to the first group with long odontostyles recovered from Arctic polar deserts.

## Materials and methods

Soil samples were collected by Dr Olga Makarova (Institute for Problems of Ecology and Evolution, Russia)from two arctic regions, i.e. Bol’shevik Island, Severnaya Zemlya Archipelago, representing a zonal type of landscape (polygonal polar desert) and the highlands of Putorana Plateau, southern Taymyr, representing an altitudinal analogue of the zonal polar deserts, i.e. a nival desert. Nematodes were extracted from 1–3 g of soil by using a Baerman funnel method for 48 hours exposition, killed by gentle heat and fixed in 4% formalin.

Nematodes were processed in anhydrous glycerin by a Seinhorst method (1959) and mounted on permanent slides. Drawings and photographs were taken using an Olympus BX51 compound microscope. Images were taken with a ColorView IIIu camera and Cell^P software (Olympus Soft Imaging Solutions Gmbh). Measurements were made using an Olympus BX 41 light microscope with a drawing tube and digitizing tablet (CalComp Drawing Board III, GTCO CalCom Peripherals, Scottsdale, AZ, USA) and Digitrak 1.0f computer program (Philip Smith, John Hutton Institute), Dundee, UK). Identification key was performed by DELTA-package software (Dallwitz 1974).

## Taxonomy

### Enchodelus makarovae sp. n.

urn:lsid:zoobank.org:act:FFC630CE-A71F-4361-A611-8CA1862AE381

http://species-id.net/wiki/Enchodelus_makarovae

**Figure 1.**
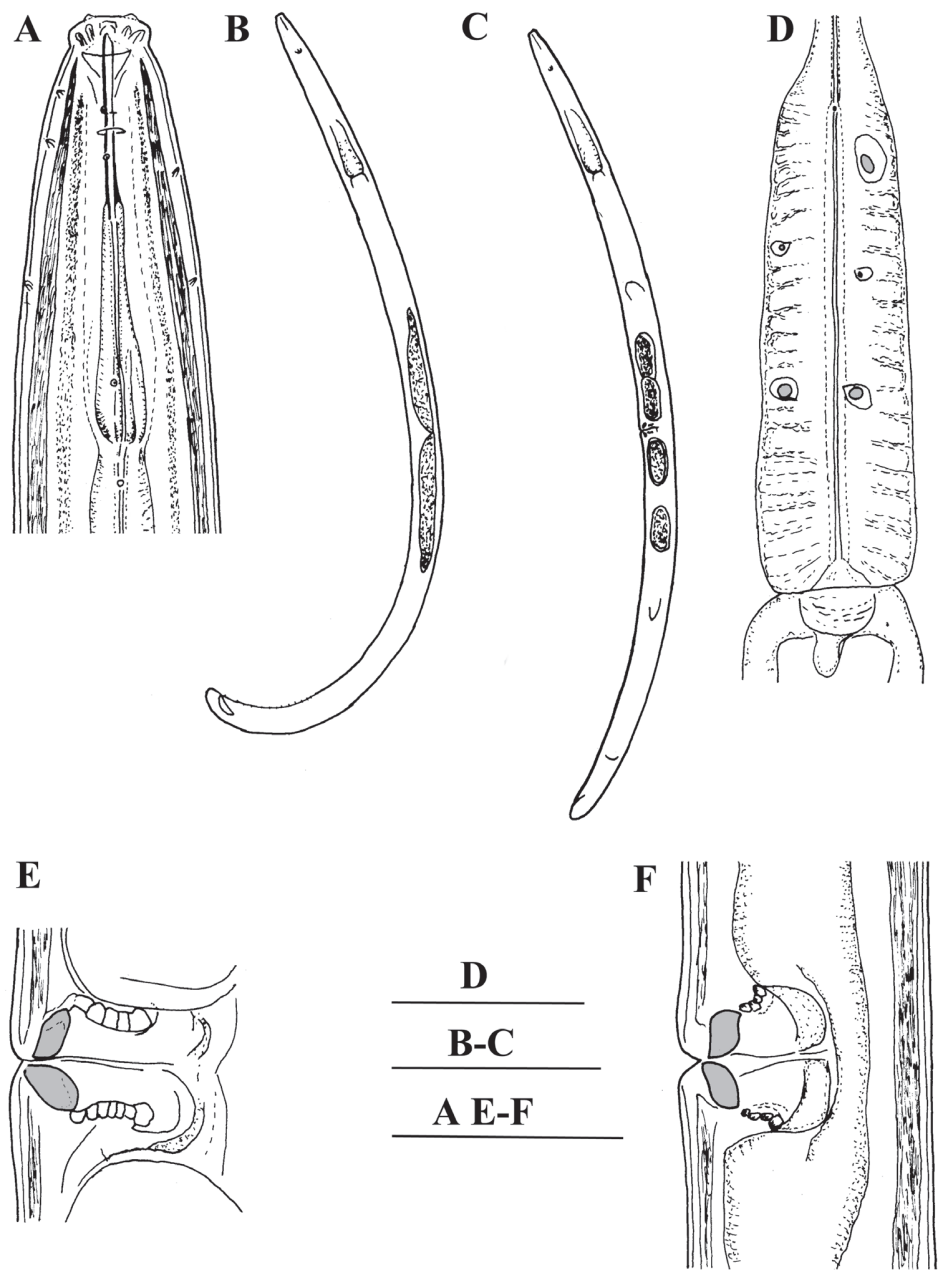
*Enchodelus makarovae* sp. n. *Female*: **A** Anterior region **C** Entire body **D** Pharyngeal bulb, dorsal and ventrosublateral glands **E, F** Vulval region. *Male*: **B** Entire body. Scale bars: **A, D, E, F** 50 µm; **B, C** 0.5 mm.

Figs 1 [Fig F2] [Fig F3] [Fig F4] [Fig F5] 6

#### Material examined.

Eight females, six males and two first stage juveniles collected from Bol’shevik Island, Severnaya Zemlya Archipelago, Russian Arctic (Table 1).

#### Measurements.

See Table 2.

#### Description.

*Female*. Body slightly ventrally curved after fixation, rarely adopting an open C shape. Cuticle smooth when viewed under light microscopy, composed of several layers with optically different appearance. Cuticle 2–4 µm thick at postlabial region, 2–3 µm - at mid body and 8–11 µm on tail, posterior to anus. Subcuticle clearly striated. Lateral chord 6–9 µm wide, occupying10–12 % of mid body diam. Lip region with slightly angular appearance, offset by depression, 2.3–3.1 times as broad as high. Labial and cephalic papillae distinct. Amphid duplex, amphidial fovea cup-shaped, opening at level of depression. Cheilostom almost cylindrical with a narrower mid-section. Odontostyle 2.3–2.8 times longer than lip region diam. or 2.0–2.6% of total body length. Odontophore 1.2–1.4 times as long as odontostyle, with flanges. Guiding ring double, located at 1.4–2.0 lip region diam. from anterior end, collar (distance between the first and second guiding ring) 3 μm. Pharynx attains the full width at 65–70% of its length from anterior end. Pharyngeal expansion 113–130 µm long or 32–37% of total pharynx length. Pharyngeal characters are presented at Table 3. Nuclei of dorsal glands 4.5–5 μm diam. and ventrosublateral 1 μm and 3–4 μm of the first and second pair, respectively. Cardia small, rounded to elongate conoid. Genital system amphidelphic, both branches almost equally developed, anterior 264–310 µm, posterior 240–310 µm. Ovaries large, 206–218 µm long; oocytes first in two or more rows, then in one row. Oviduct 168–172 µm long, 2.1–2.4 times body diam., *pars dilatata oviductus* well developed. Sphincter between oviduct and uterus distinct. Uteri long, anterior and posterior uterus with almost equal length (267.6±56.3 (220–346) μm, n=5 and 284.0±25.5 (256–332) μm, n=6), or 2.9–4.9 times corresponding body diam. Uterus tripartite, consisting of a wider proximal portion with distinct lumen (146 μm, n=1), followed by a slender median portion (118, 112 μm, n=2) and ending with a well developed spheroid *pars dilatata distalis uteri*. Vagina extending inwards 27–42 μm or 38–59% of body diam., *pars proximalis* 24x26μm (n=1), *pars refringens* with two trapezoid sclerotisations, with a combined width of 20–21μm and length 6–8μm (n=2),* pars distalis* 5–7 μm, n=4. Two females with 3 and 4 uterine eggs, respectively, measuring 37–45 × 98–106 µm. Prerectum variable in length, 2.1–3.5 times the anal body width; rectum 0.6–1.1 anal body diam. long. Tail bluntly conoid with elongated saccate bodies present mostly along ventral side. Hyaline part of tail 8–12 µm thick or 24–47 % of total tail length. Two pairs of subterminal caudal pores, one subdorsal, another lateral.

*Male*. General morphology similar to that of the female, except for genital structures. Arrangement of pharyngeal gland nuclei is presented at Table 3. Lateral chord very narrow (4–6 μm) occupying10–12 % of mid body diam. with scattered glandular bodies. Reproductive system diorchic, composed of two opposed testes, anterior 311, 319 µm (n=2) and posterior 275, 285 µm (n=2) long. Sperm cells spindle-shaped, measuring 6–9 × 2 µm. Spicules dorylaimoid, 1.5–1.7 times anal diam. long, lateral accessory pieces paired, more or less cylindrical with bifurcate end, measuring 16–18 × 3 µm (n=2). Ventromedian supplements 10–12 in number preceded by one adcloacal pair of papillae located at 8–11 µm apart from cloacal opening, 0–1 in the range of spicules; moderately developed postcloacal papilla present. Prerectum 3.3–4.0 anal body diam. long. Tail bluntly conoid, ventrally almost straight, dorsally convex with broadly rounded terminus, two pair of caudal pores.

*Juveniles*. Two first stage juveniles were recovered. Body almost straight. Lip region flat, continuous with the body, genital primordium 11 μm long, tail conical with long central peg, 30, 33 μm long.

#### Diagnosis and relationships.

The new species *Enchodelus makarovae* sp. n. is an amphimictic species distinguished by females with body length of 1.57–2 mm, lip region 15–17.5 µm wide, amphid duplex, odontostyle 38–43 µm long or 2.3–2.8 times lip region diam. Odontophore with flanges, 1.2–1.4 times as long as odontostyle, pharynx length 320–377 µm, pharyngeal expansion 113–130 µm long or 32–37% of total pharynx length, female genital system amphidelphic, uterus tripartite, *pars refringens vaginae* with two trapezoid sclerotisations, vulva transverse slit, V=45–51%, tail rounded conoid (25–35 µm, c=45.8–70.3, c’=0.6–0.9 in females, and 29–33 µm, c=46.4–58.9, c’=0.7–0.8 in males). Males with 65–74 µm long spicules and 10–12 spaced ventromedian supplements.

Based on tail morphology and odontostyle length this species can be assigned to the *Enchodelus macrodorus* – group as defined by Guerrero et al. (2008). Thisgroup includes *Enchodelus babakicus* Pedram et al. 2009, *Enchodelus carpaticus* Ciobanu et al., 2010, *Enchodelus distinctus* Ahmad & Jairajpuri, 1980, *Enchodelus groenlandicus* (Ditlevsen, 1927) Thorne, 1939, *Enchodelus macrodorus* (de Man, 1880) Thorne, 1939, *Enchodelus microdoroides* Baqri & Jairajpuri, 1974 and *Enchodelus saxifragae* Popovici, 1995. This homogeneous group is characterised by the presence of a rather long odontostyle (>35µm), odontophore with well developed flanges, uterus tripartite (except for *Enchodelus distinctus*, which has been described with a bipartite uterus (Ahmad and Jairajpuri 1980) and hemispheroid to rounded conoid tail.

In having a lip region set off by a depression the new species is most similar to *Enchodelus carpaticus*, *Enchodelus groenlandicus*, *Enchodelus macrodorus* and *Enchodelus microdoroides*. However, it can be separated from *Enchodelus carpaticus* by its shorter pharyngeal expansion (113–130 *vs* 136–167 µm), different arrangement of pharyngeal glands, DN and S2N situated more posteriorly (DN=69–72% *vs* DN=63–65%, S2N=86–89% *vs* SN=82–86 %, respectively), absence of dorsal cell mass near cardia *vs* presence, ovaries large (206–218 µm long) *vs* short (61–155 µm long), prerectum shorter (87–140 *vs* 164–272 μm or 2.1–3.5 *vs* 4.1–6.6 anal body diam), saccate bodies present *vs* absent, males abundant *vs* absent (in *Enchodelus carpaticus* males not found, but sperm cells were observed in one female from a Romanian population (Ciobanu et al. 2010)); it should be mentioned also that there are differences in average values of odontostyle (av. 40.7 (38–43 µm) *vs* av. 43.3 (39.5–47 µm) and tail length (av. 29.2 (25–35) *vs* av. 23.7 (21–29) μm), and c’ value (c’= av. 0.7 (0.6–0.9) *vs* av. 0.6 (0.5–0.7); from *Enchodelus groenlandicus* by its shorter odontostyle (38–43 *vs* 44–53 µm), somewhat more anteriorly located guiding ring (24–28 *vs* 27–37 µm), narrower lip region (15–17.5 *vs* 19–22 µm), males present *vs* absent; from *Enchodelus macrodorus* this new species differs in having a longer ovarium and oviduct (206–218 *vs* 83–188 μm and 168–172 *vs* 97–159 μm, respectively (Thorne’s specimens), longer uterus (220–346 *vs* 61–143 and 56–115 μm) and shorter prerectum (2.1–3.5 *vs* 3.9–5.8 anal body diam), tail somewhat longer (25–35 *vs* 18–24 and 22–28 μm) and differently shaped (bluntly conoid *vs* rounded to hemispherical), saccate bodies large elongated *vs* small round; males abundant *vs* males rare; longer tail in males (29–33 *vs* 18–22 μm, c=46.4–58.9 *vs* 67–100 and c’=0.7–0.8 *vs* 0.6) (Guerrero et al. 2007b, 2008); from *Enchodelus microdoroides* by its longer body in females (1.57–2 *vs* 0.94–1.29 mm), wider lip region (15–17.5 *vs* 13–14 µm),guiding ring located more anteriorly (24–28 *vs* 28–39 µm from anterior end), different shape of *pars refringens vaginae* (trapezoid *vs* rectangular), longer tail (25–35 *vs* 13–27 µm) and males with longer spicules (65–74 *vs* 45–50 µm).

The new species can be distinguished from the remaining three species of *Enchodelus macrodorus* group by its lip differentiation: lip region set off by depression *vs* offset by a distinct constriction. Further, it differs from *Enchodelus babakicus* by its longer body in female (1.57–2 *vs* 1.21–1.56 mm), ovaries longer (206–218 µm *vs* 39–63 µm), longer uterus (220–346 vs 130–175 μm) and tail (25–30 vs 16–22 μm); shorter prerectum (87–140 *vs* 151–232 μm or 2.1–3.5 *vs* 4.5–8.5 anal body diam. long), males with longer spicules (65–74 *vs* 49–61 µm) and narrower lateral chord (10–12 vs 15–20% of corresponding body diam.); different tail shape in first stage juvenile (straight *vs* ventrally curved); from *Enchodelus distinctus* the new species is differentiated by its longer odontostyle (38–43 *vs* 36 µm), more posteriorly located guiding ring (24–28 *vs* 21–23 µm), different structure of uterus (tripartite *vs* bipartite), saccate bodies present *vs* absent. Finally, the new species can be distinguished from *Enchodelus saxifragae* by a narrower lip region (15–17.5 *vs* 18–22 µm or 2.3–2.8 *vs* 1.8–2.3 odontostyle as lip region diam.), shorter pharyngeal expansion (av.121 (113–130) *vs* av.153 (144–162.5) and av. 147 (116–186) µm), shorter prerectum (87–140 *vs* 140–294 μm or 2.1–3.5 *vs* 4–8 anal body diam ) and fewer ventromedian supplements (10–12 *vs* 13–16) (Popovici 1995, Guerrero et al. 2008).

#### Type-locality and habitat.

Different types of vegetation from a polygonal polar desert on Bol’shevik Island, Severnaya Zemlya Archipelago, Russian Arctic (Table 1).

#### Type-material.

Holotype, 5 paratype females, 4 paratype males and 2 juveniles deposited in the Nematode collection of the Institute of Biodiversity and Ecosystem Research, BAS; one female and one male paratypes each at the nematode collections of the following institutions: The Center of Parasitology of Institute for Problems of Ecology and Evolution, RAS, Russia and Plant Protection Service, Wageningen, The Netherlands.

#### Etymology.

The species is named in honor of Dr. Olga Makarova (Institute for Problems of Ecology and Evolution, Russia) who is an outstanding biologist investigating polar habitats and has kindly provided us with numerous nematode materials from Arctic polar deserts.

**Figure 2. F2:**
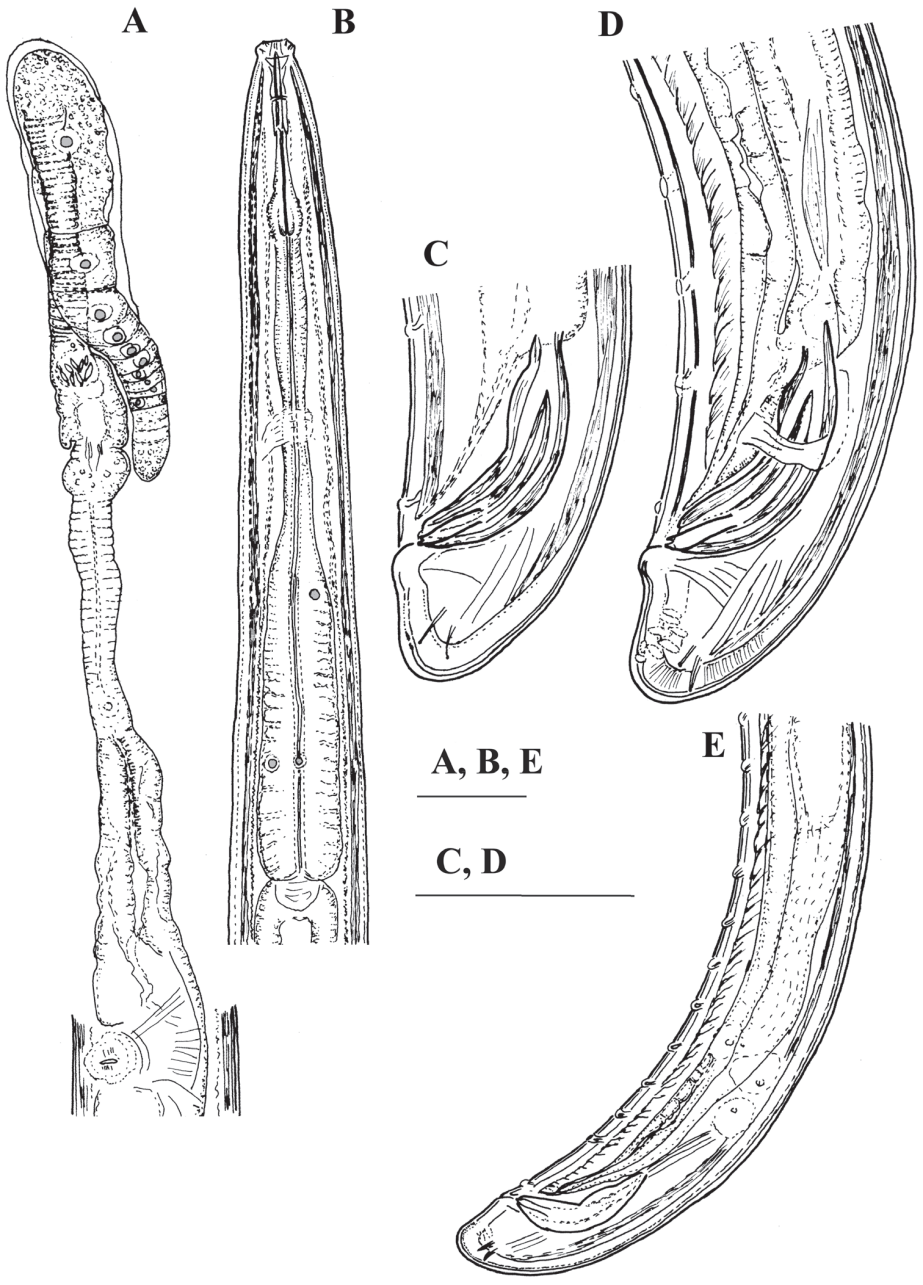
*Enchodelus makarovae* sp. n. **A, B** *Female*: **A** Anterior genital branch **B** Neck region *Male*: **C,** **D, E** Posterior ends. Scale bars: **A–E** 50 µm.

**Figure 3. F3:**
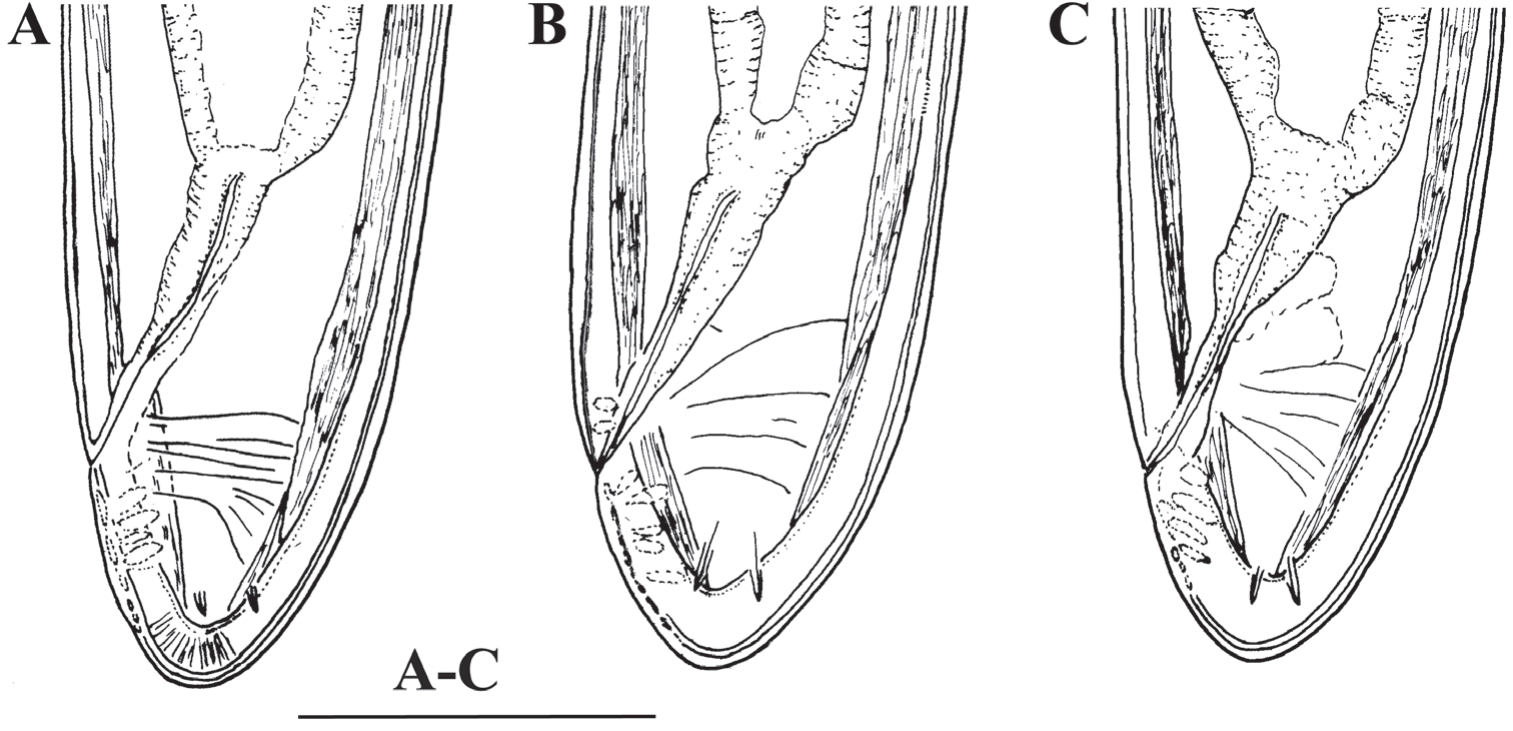
*Enchodelus makarovae* sp. n. **A–C** *Female*: Variability of female tail. Scale bars: **A–C** 50 µm.

**Figure 4. F4:**
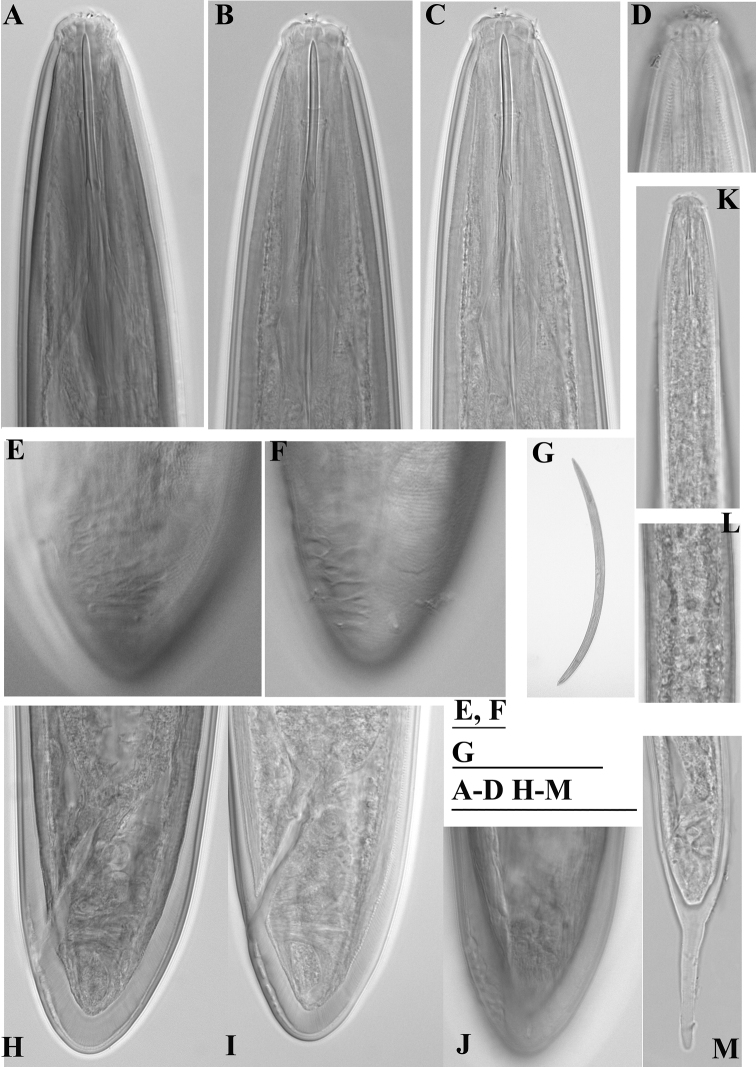
*Enchodelus makarovae* sp. n. **A–J** *Female*: **A–C** Variability of anterior region **D** Amphidial fovea **G** Entire body **E, F, J** Variability of tail with saccate bodies **H, I** Tail end **K–M** *Juveniles* **K** Anterior region **L** Genital primordium **M** Tail. Scale bars: **A–D H–M** 50 µm; **G** 1 mm, **E, F** 10 µm.

**Figure 5. F5:**
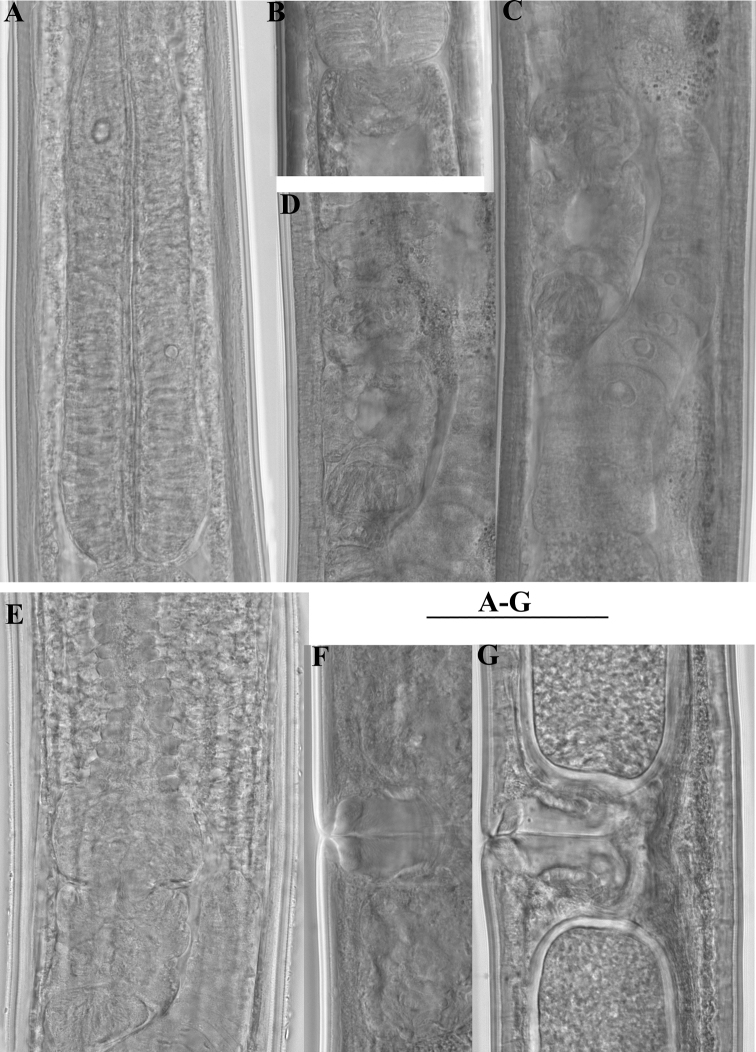
*Enchodelus makarovae* sp. n. *Female* **A** Pharyngeal bulb, dorsal and ventrosublateral glands **B **Cardia **C, D** *Pars dilatata oviductus* and ovarium **E** *Pars dilatata distalis uteri* **F–G** Vulval region. Scale bars: **A–G** 50 µm.

**Figure 6.**
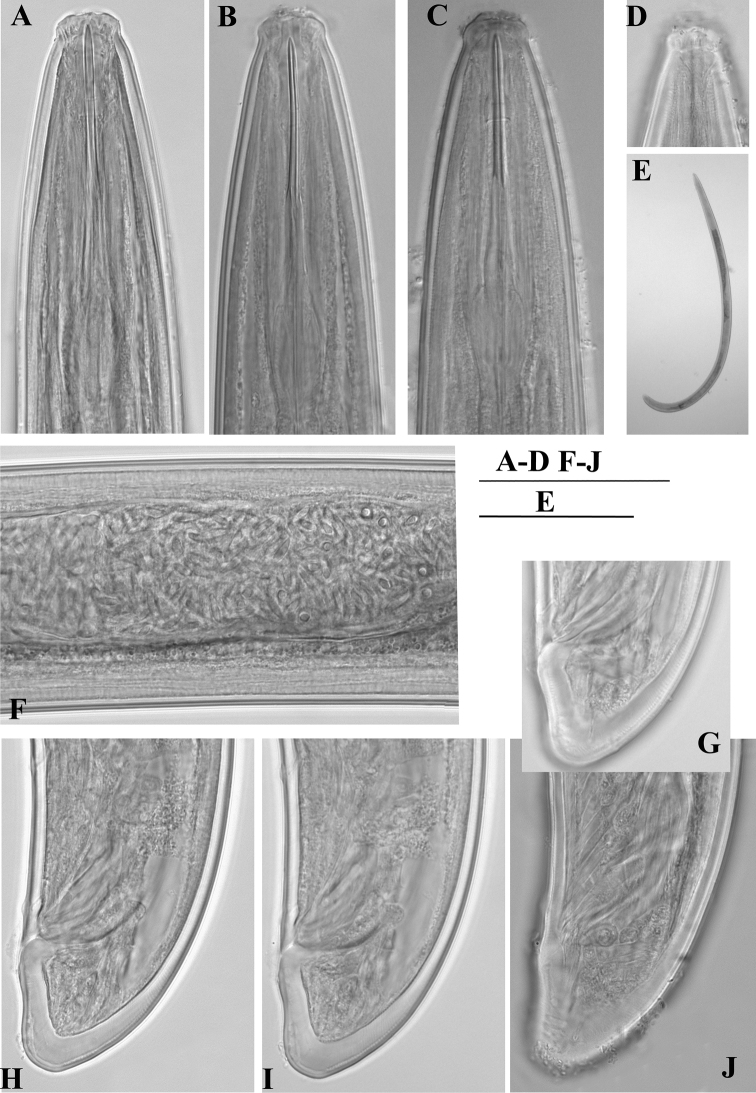
*Enchodelus makarovae* sp. n. **A–J** *Male*: **A–C** Anterior ends **D** Amphidial fovea **E** Entire body **F** Sperm cells in testis **G** Lateral piece **H, I** Tail ends **J** Tail with saccate bodies. Scale bars: **A–D, F–J** 50 µm; **E** 1 mm.

**Table 1.** Distribution of *Enchodelus makarovae* sp. n. and *Enchodelus groenlandicus* in Arctic polar deserts.

Locality and samples	Type of landscape and vegetation	Abbreviation	Nematode species
Bol’shevik Island Severnaya Zemlya Archipelago 78°12'N, 103°17'E	Polygonal polar desert		*Enchodelus makarovae* sp. n.
Site 1Collected on 09.08.1997			
Sample № 2	*Alopecurus alpinus* Sm.	AA	3♀ 1♂
Sample № 3	*Gymnomitrium coraloides* Nees.	GC	1♀ 1♂
Site 2Collected on 13.08.2000			
Samples № 6, 8 and 9	*Gymnomitrium coraloides* and *Lopadium* sp.	GC & L	4♀ 3♂
Sample № 13	Black crust with a small tuft *Deshampsia borealis* (Trautv.) Roshev.	DB	1♂
Sample № 7	Black crust	BC	2 J_1_
Putorana Plateau Taymyr Peninsula69°09'N, 91°52'E	Polygonal nival desert		*Enchodelus groenlandicus*
750 m a.s.lCollected on 3.08.1996			
Sample № 7	Old *Deshampsia borealis* tuft with *Gymnomitrium corralioides* and *Cladonia* sp.	DB, GC, C	3♀
Samples № 9 and 10	Large green *Deshampsia borealis* tuft	DB_1_	7♀

**Table 2.** Morphometrics of *Enchodelus makarovae* sp. n. from Bol’shevik Island, Severnaya Zemlya. All measurements, unless indicated otherwise, are in µm.

Characters		GC&L		GC		AA		DB	Range		BC
	Holotype	Female	Male	Female	Male	Female	Male	Male	Femalen=8	Malen=6	_J_1n=2
L (mm)	2.00	1.76, 1.79, 1.62	1.70; 1.67; 1.79	1.85	1.71	1.64,1.57,1.85	1.77	1.49	1.76±0.1(1.57-2.00)	1.69±0.1(1.49-1.79)	0.62, 0.53
a	28.3	33.1, 25.6, 21.6	28.1, 26.5, 29.2	24.2	29.8	22.6,23.1, 23.1	23	19.6	25.2±3.8(21.6-33.1)	26.0±3.9(19.6-29.8)	24.7, 25.7
b	5.6	5.7, 5.3, 4.4	5.4, 4.4, 5.3	4.9	4.9	5.1, 4.7, 5.3	5.2	4.5	5.1±0.4(4.4-5.7)	4.9±0.4(4.4-5.4)	3.9, 3.4
c	70.3	69.9, 60.5, 45.8	58.9, 54.9, 55.8	59.7	51.7	54.1, 49.6, 61.2	53.0	46.4	61.1±7.6(45.8-70.3)	53.4±4.2(46.4-58.9)	11.4, 9.8
c‘	0.6	0.6, 0.7, 0.9	0.7, 0.7, 0.8	0.7	0.8	0.8, 0.9, 0.6	0.7	0.8	0.7±0.1(0.6-0.9)	0.7±0.1(0.7-0.8)	2.9, 3.1
V %	51	49, 51, 48	-	49	-	48, 48, 45	-	-	48.6±1.8(45-51)		-
Lip region width	17	17, 17, 16	16, 17, 17.5	17.5	16	17, 16, 15	17	16	16.6±0.8(15-17.5)	16.7±0.5(16-18)	9, 9
Odontostyle	40	41, 41.5, 38	44, 39, 44	43	44	38.5, 42, 41	44.5	43	40.7±1.6(38-43)	42.9±2.1(39-44.5)	10, 9.5
Replacement odontostyle	-	-	-	-	-	-	-	-			12, 11
Odontophore	54	49, 49.5, 50	49, 50, 53	57.5	52	47, 53, 57	52	54	52.3±3.9(47-57.5)	51.3±1.6(49-54)	-
Spear	95	90, 91, 89	93, 89, 96	100.5	96	85.5, 95, 99	96	97	93±5.1(85.5-100.5)	94.3±2.9(89-97)	-
Anterior end guiding ring	26	24, 24, 24	22, 25, 28	26	27	25, 28, 28	26	28	25.7±1.8(24-28)	25.9±2.0(22-28)	5.5, 6.0
Neck length	355	320, 336, 366	318, 384, 342	377	354	321, 341, 349	340	333	345.7±20.3(320-377)	344.9±22.3(318-384)	160, 155
Width at pharynx base	63	51, 63, 69	56, 62, 58	66.5	60	66, -, 69	72	70	64.0±6.1(51-69)	62.9±6.6(56-72)	25, 22
Width at mid-body	71	54, 70, 75	61, 63, 62	77	58	73, 68, 80	77	76	70.9±8.06(54-80)	66.0±8.3(58-77)	25, 21
Prerectum length	138	87, -, 139	-, 185, 132	126	163	140,-,138	-	163	128±20.6(87-140)	160.7±21.7(132-185)	53, -
Rectum length	40	23, 47, 40	-	49	-	41, -, 42	-	-	40.3±8.3(23-49)		10, -
Tail	29	25, 30, 35	29, 31, 32	31	33	30, 32, 30	33	32	29.2±3.3(25-35)	31.7±1.6(29-33)	55, 55
Spicules	-	-	65, 73, 67	-	71	-	74	70		70.1±3.5(65-74)	
Ventromedian supplements	-	-	10, 12, 10	-	11	-	12	11		10-12	

**Table 3.** Pharyngeal characters of *Enchodelus makarovae* sp. n. and *Enchodelus groenlandicus*. For abbreviations see (*) Loof and Coomans (1970) and (**) Andrássy 1998. All data are given in percent.

	*Enchodelus makarovae* sp. n.						*Enchodelus groenlandicus*	
	Bol’shevik Island						Putorana Plateau	
Characters	GC&L		GC		AA	DB	DB1	DB, GC, C
	females	males	female	male	females	male	females	females
DN=D	69, 70,70	69, 67, 70	71	70	72, 71	69	64-71 (n=7)	62, 60
S1N1*		76	77				75-80 (n=5)	72, 70
S1N2*			78				76	
S2N1*	86, 87, 86	85, 87, 87	87		87, 88	86	85-87 (n=7)	84, 81
S2N2*	87, 87, 86	84, 87, 87	88		88, 89	86	85-88 (n=7)	84, 81
AS1**		18	21				15-35 (n=5)	26, 24
AS2**			26				17	
PS2**	55, 57, 53	53, 60, 56	55		55, 58	55	52-60 (n=7)	56, 54
PS2**	59, 56, 52	49, 59, 55	56		56, 61	54	53-60 (n=7)	58, 53

### Enchodelus groenlandicus

(Ditlevsen, 1927) Thorne, 1939

http://species-id.net/wiki/Enchodelus_groenlandicus

**Figure 7.**
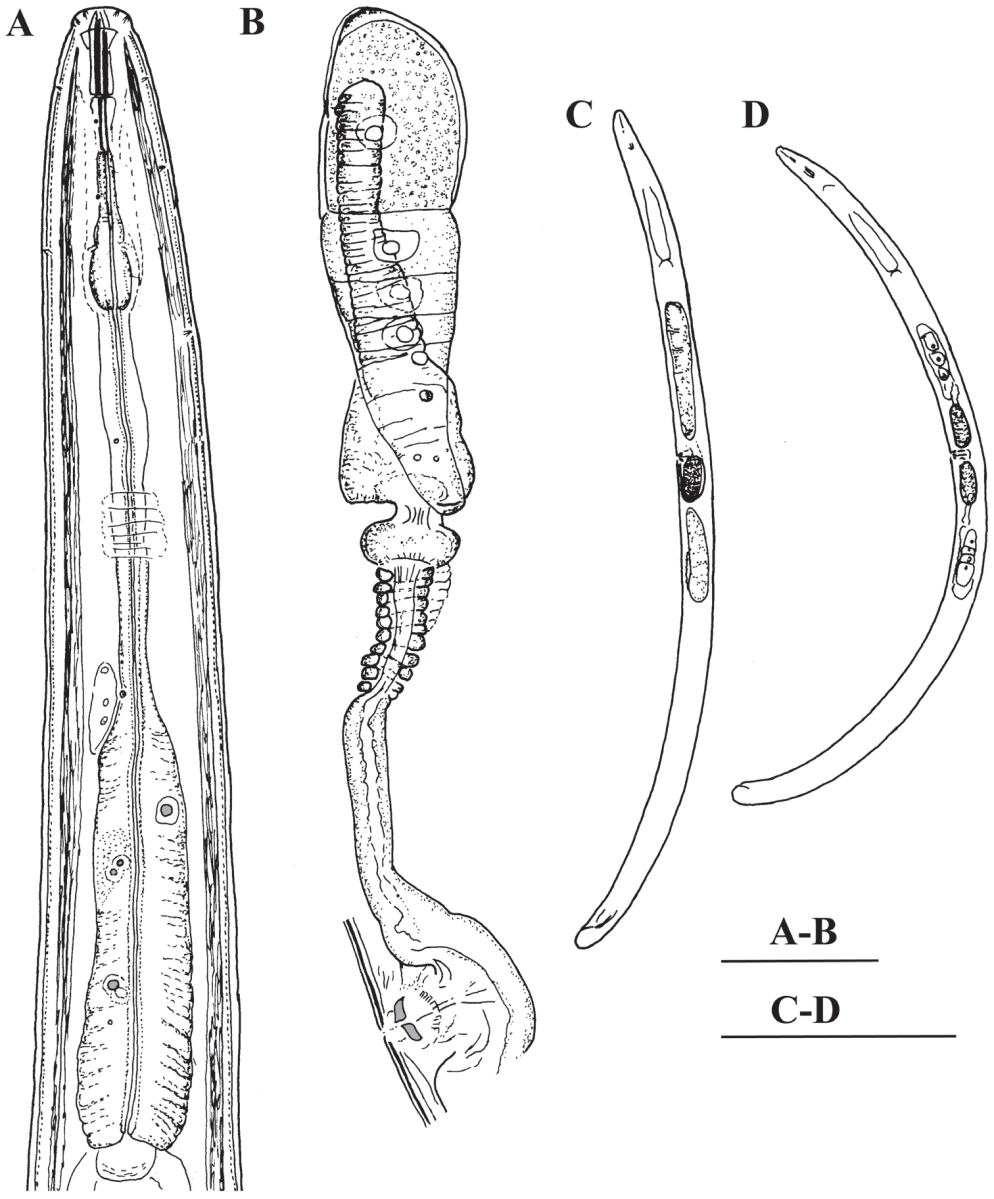
*Enchodelus groenlandicus*. **A–D** *Female* **A** Neck region **B** Anterior genital branch **C, D** Entire body. Scale bars: **A, B** 50 µm; **C, D** 500 µm.

Figs 7 [Fig F8] [Fig F9] [Fig F10] 11

#### Material examined.

Ten females collected from Putorana Plateau, Russian Arctic (Table 1).

#### Measurements.

See Table 4.

#### Description.

*Female*. Nematodes of medium to large size, habitus from slightly curved ventrad to open C- shape after fixation. Cuticle with fine, but distinct transverse striations, especially visible at neck and on tail regions; 4–6 µm thick at postlabial region, 3–4 µm at mid-body and 7–8 µm on tail. Lateral chord narrow, 6–9 µm wide or occupying *ca* 9–13 % of mid body diam. Lip region rounded, offset by a depression, 2.3–3.1 times as wide as high. Amphidial fovea cup-shaped, located at level of labial depression, occupying 65% of lip diam. Cheilostom cylindrical. Odontostyle long, 2–2.5 times longer than lip region diam. or 2.2–2.7% of total body length. Odontophore distinctly flanged, 1.1–1.3 times as long as odontostyle. Guiding ring double, located 1.4–1.6 lip region diam. from anterior end. Pharynx attains full width at 56–64% of its length from anterior end. Pharyngeal characters are presented at Table 3. Cardia rounded measuring 6–10 × 15–17 µm. Genital system amphidelphic, both branches equally and well developed, anterior 277–370 µm, posterior 287–375 µm long. Ovaries relatively large, 142–303 µm long; oocytes firstly in two or more rows, then in a single row. Anterior and posterior oviduct 119–143 µm (n=9) and 119–153 µm (n=8) long, 1.6–1.9 and 1.6–2.0 times body diam. respectively, consisting of slender part and well developed *pars dilatata oviductus*. Sphincter distinct. Uterus thick walled, tripartite, consisting of a wider proximal portion with distinct lumen, followed by a narrower median portion (43–115 µm (n=4)) and ending with a well developed spheroid *pars dilatata distalis*. Vagina extending inwards 42–53 *μ*m or 53–68 % of body diam., *pars proximalis* 25–30 µm × 21–22 µm,* pars refringens* with two trapezoid sclerotisations, with a combined width of 18–18.5μm and length 6–8 μm(n=2) *pars distalis* 6 µm long (n=2). Vulva a transverse slit, pre-equatorial (40–47%). Eggs observed in eight females, measuring 96–109 × 43–64 µm, most frequently located in *pars dilatata oviductus* (n=6), rarely in uterus (n=2). Prerectum 3–4.5 anal diam. long, rectum 0.8–1.0 times anal body width. Tail hemispheroid. Numerous small elongated saccate bodies observed on tail, mostly on ventral side. Hyaline part of tail 8.0–10 µm thick or 25–33 % of total tail length.

*Male*. Unknown

#### Locality and plant associations.

Different types of vegetation from a polygonal polar desert on Plateau Putorana, Russian Arctic (Table 1).

#### Remarks.

The data on *Enchodelus groenlandicus* geographical distribution, *i.e*. the original description from Disko Island, Greenland (Ditlevsen 1927) and recent reports from Spain, Albania and Iran (Guerrero et al. 2008, Andrássy 2009b, Pedram et al. 2011) indicate a disjunctive type of range. It occurs at high altitudes 950 m to 2450 m a.s.l in Southern Europe and Iran, and at high latitudes in the polar region (Putorana Plateau and Greenland), Guerrero et al. (2008) hypothesize that such a distribution pattern might stem from quaternary glacial events. The specimens examined generally agree well with data reported for this species, although some differences occurred: the Arctic population has somewhat shorter odontostyles (43–47 *vs* 44–53 µm) and a more anterior position of the vulva (40–46 *vs* 41.5–49.4%, see Table. 4 for details); Iranian specimens had shorter female tails (19–24 *vs* 28–33 *μ*m and c’= 0.4–0.6 *vs* 0.5–0.7). We consider the morphometric differences as representing intraspecific variation.

**Figure 8. F8:**
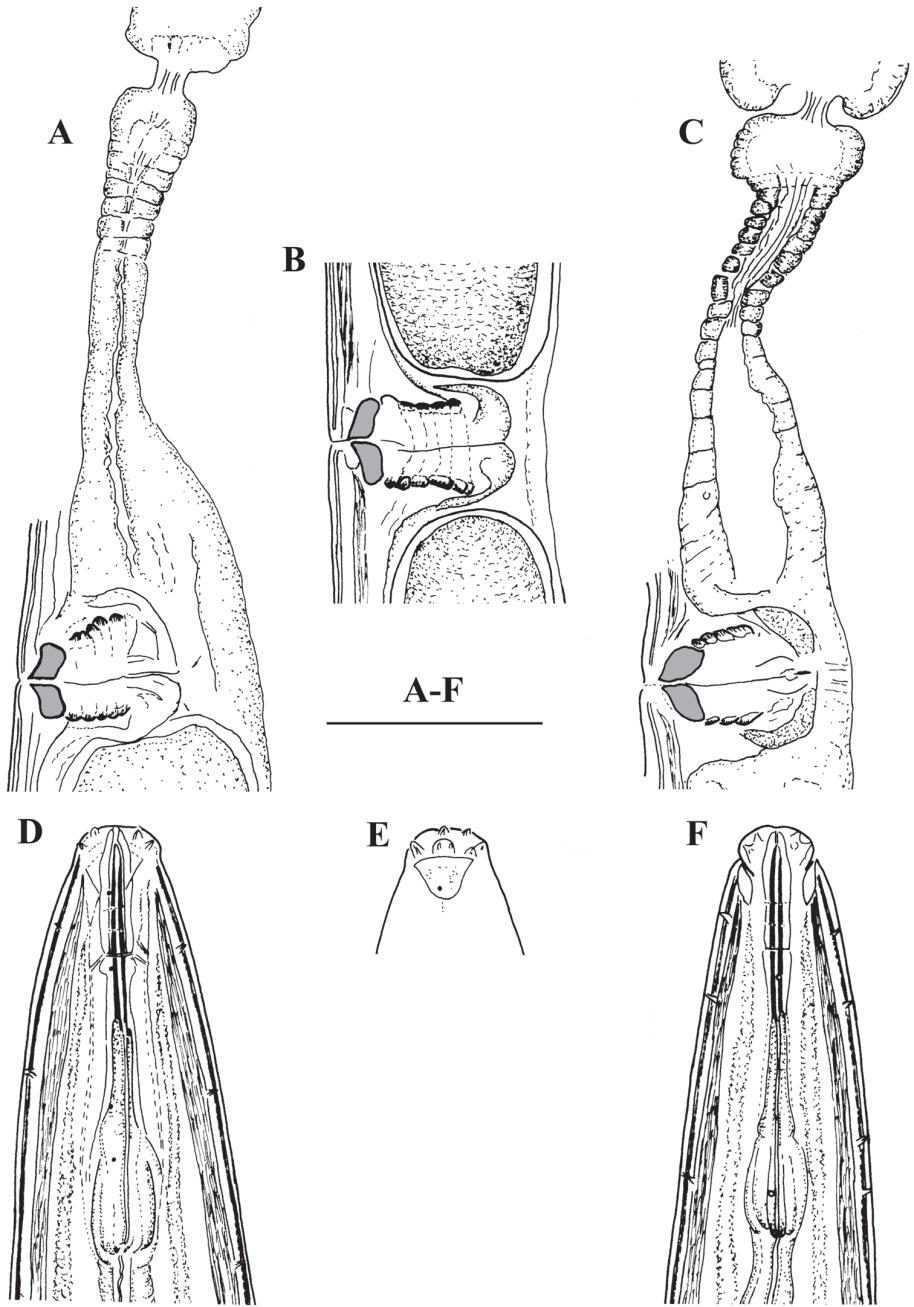
*Enchodelus groenlandicus*. **A–F** *Female* **A, C** Vulval region and uterus **B** Vulval region **D  **Anterior region, lateral view **E** Amphidial fovea **F** Anterior region, ventral view. Scale bars: **A–F** 50 µm.

**Figure 9. F9:**
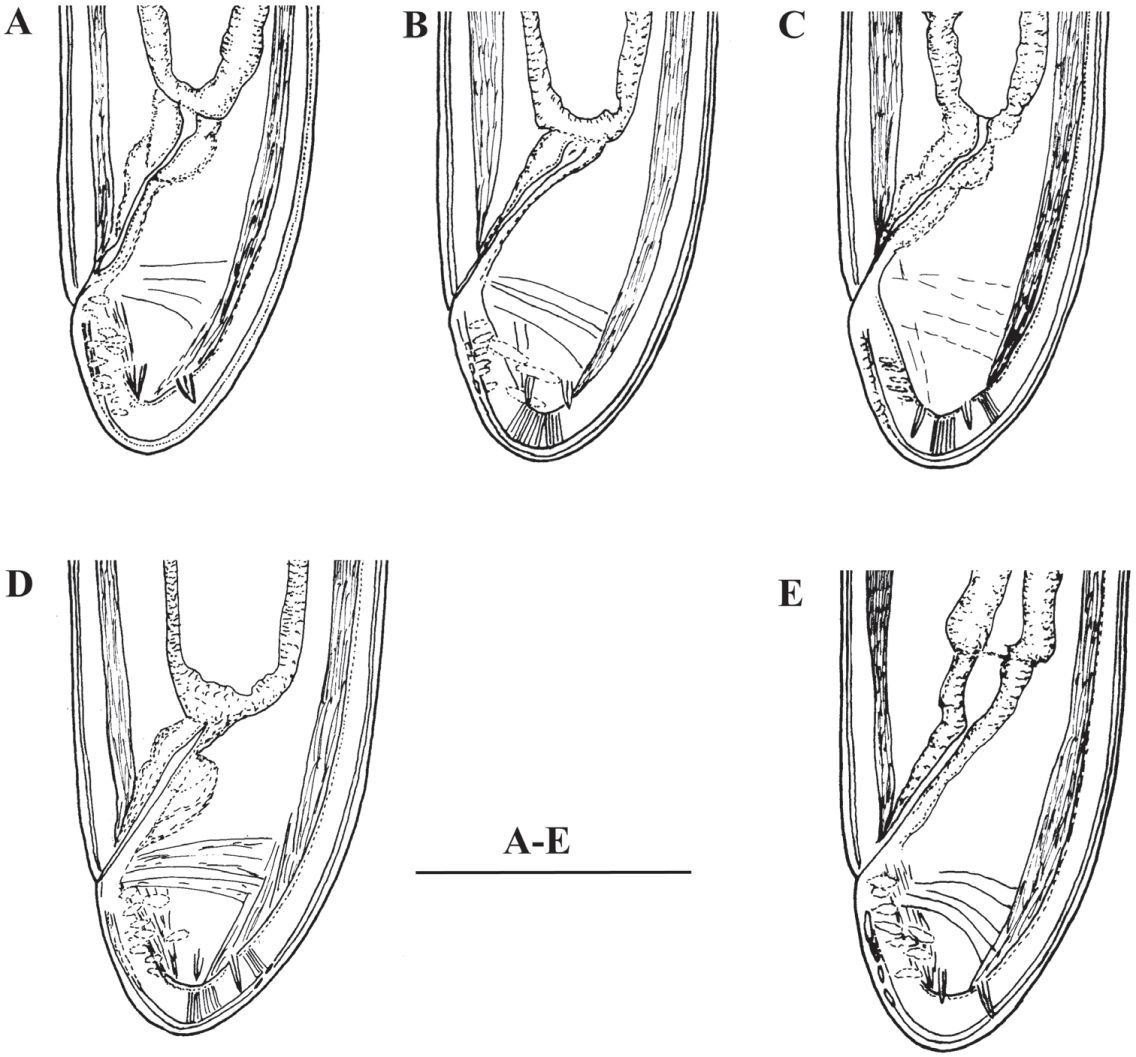
*Enchodelus groenlandicus*. **A–E** *Female* **A–E** Tail ends. Scale bars: **A–E** 50 µm.

**Figure 10. F10:**
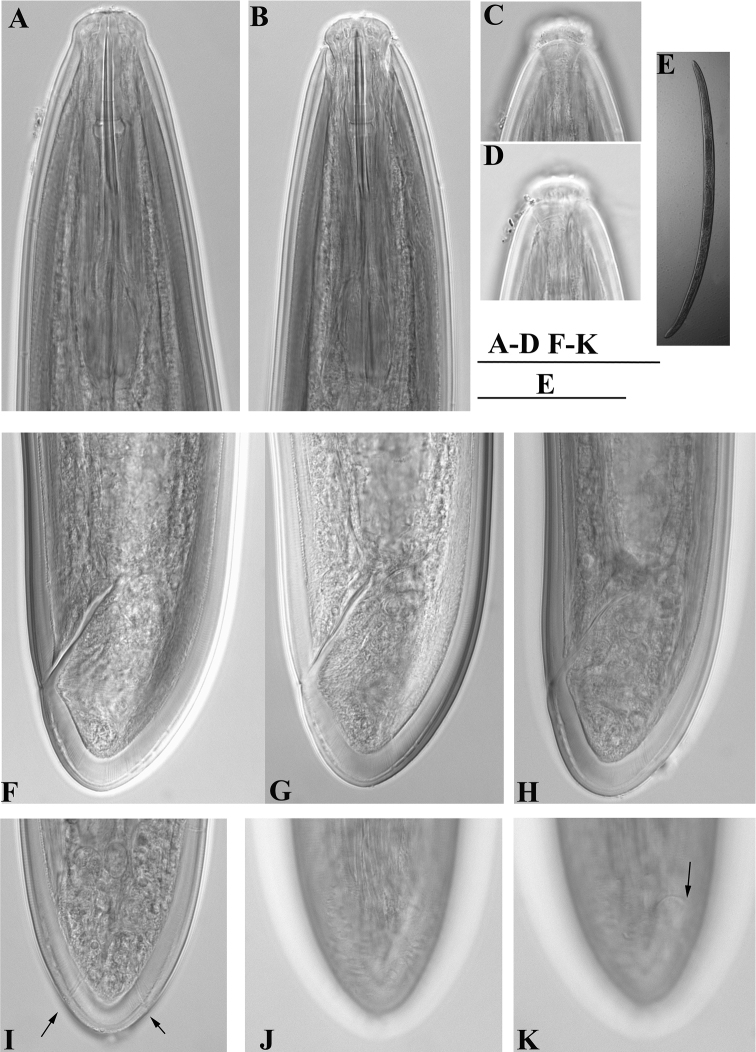
*Enchodelus groenlandicus*. **A–K** *Female*:**A, B** Anterior region **C, D** Amphideal fovea **E** Entire body **F–H** Tail ends **I** Subterminal caudal pores indicated by arrows **J** Tail with saccate bodies **K** Tail in ventral view, anus marked by an arrow. Scale bars: **A–D, F–K** 50 µm; **E** 1 mm.

**Figure 11.**
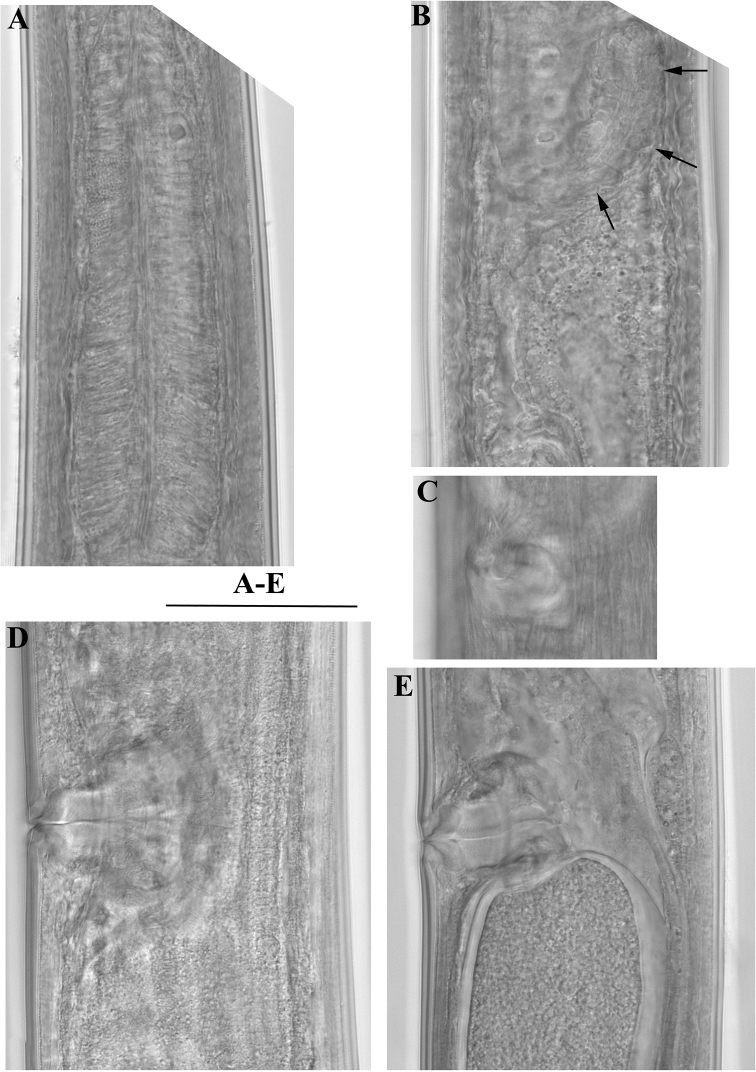
*Enchodelus groenlandicus*. **A–E** *Female*: **A** Pharyngeal bulb **B** *Pars dilatata distalis uteri* (arrows) **C** Vulval region in subventral view **D, E** Vulval region in lateral view. Scale bars: **A–E** 50 µm.

**Table 4.** Morphometrics for females of *Enchodelus groenlandicus* (Ditlevsen, 1927) Thorne, 1939. All measurements, unless indicated otherwise, are in µm (and in the form: mean±SD (range).

Characters	Russia – Putorana Plateau		Greenland	Spain	Albania	Iran
	Present study		Ditlevsen 1927	Guerrero et al. 2008	Andrássy 2009	Pedram et al. 2011
	DB_1_	DB, GC, C				
n	7	3	1*	14	2	4
L (mm)	1.94±0.16(1.8-2.16)	1.77, 1.70, 1.92	2.5	1.78±0.15(1.57-2.07)	1.54-1.68	1.86±0.09(1.76-1.97)
a	24.4±1.8(21.7-25.9)	24.3, 25.9,	25	23.4±1.6(21.3-25.3)	22-23	23.0±2.5(20.3-26.0)
b	5.3±0.3(5-5.6)	4.6, 4.7, 4.8	6	5.1±0.3(4.5-5.5)	4.0-4.6	5.0±0.2(4.6-5.1)
c	64.9±4.9(59.9-70.8)	61.5, 52.4, 62.1	50	67.5±9.2(53-83)	40-46	85.5±14.0(73-104)
c’	0.6±0.1(0.5-0.7)	0.6, 0.7, 0.7	0.7	0.7±0.1(0.6-0.8)	0.7-0.8	0.5±0.1(0.4-0.6)
V %	42.4±1.8(40-44)	46, 43, 42	43	44.2±1.9(41.6-49.4)	44-45	42.5±1.0(41.5-44.0)
Lip region width	19.8±0.8(19-20.5)	21, 21, 19	20	20.5±0.9(19-22)	19-20	22.5±0.5(21-23)
Odontostyle	46.7±0.4(46-47)	47, 44, 43	48-49	49.3±2.3(44-53)	50-51	48.5±0.5(48-49)
Odontophore	49±0.2(48.7-49)	50, 48, 55	49	50.4±2.9(45-55)	52-54	52±1(51-53)
Spear	95.6±0.3(95-96)	97.5, 93, 98	98	100±4.1(94-108)	102-106	102.5±1.0(101-103)
Anterior end to guiding ring	30.4±1.9(29-33)	30, 28, 30	29	32.8±2.4(27-37)	-	-
Neck length	376.6±12.4(361-398)	389, 361, 398	417	354±24.0(322-401)	-	377.5±19.0(350-392)
Width at pharynx base	68.5±3.9(65-75)	69, 60, 62	-	66.0±9.0(49-75)	-	78.5±5.0(75-82)
Width at mid body	79.5±4.1(75.5-83)	73, 66, 68	100	76.1±5.5(67-87)	77-80	82.0±9.5(68-89)
Prerectum length	185.6±10.6(178-193)	187,-, 213	50	186±36(116-252)	-	203.0±19.5(176-223)
Rectum length	42.1±2.5(39-44.5)	38, 46, 42	-	42.1±7.3(27-52)	-	-
Tail	29.9±1.7(28-32)	29, 33, 31	31	26.8±4.2(22-37)	28-30	22.0±2.5(19-24)

*followed by Guerrero et al. 2008

##### Identification key to species belonging to *Enchodelus macrodorus* group

**Table:** 

1	Odontostyle ≤ 36 µm; uterus bipartite (♀ L=1.85 mm, a=20, b=5.1, c=58, c’=0.76, V=53%, Odontostyle=36 μm; ♂ unknown) (India)	*Enchodelus distinctus* (Ahmad & Jairajpuri, 1980)
–	Odontostyle ≥37 µm; uterus tripartite	2
2	Lip region separated by constriction	3
–	Lip region separated by depression	4
3	Body long (>1.6 mm) (♀ L=1.8–2.38 mm, a=21–34, b=4.8–6.1, c=54–92, c’=0.6–0.9, V=44–50%, Odontostyle=38–45 µm; ♂ L=1.66–2.21mm a=24.4–32.7, b=5.1–5.9, c=53.2–68, c’=0.7–0.8, Odontostyle=27.5–40 µm, Spicules= 56–70 µm) (Romania, Spain)	*Enchodelus saxifragae* (Popovici, 1995)
–	Body short (<1.6 mm) (♀ L=1.21–1.56 mm, a=20–25.5, b=3.0–5.0, c=60.5–92.5, c’=0.5–0.7, V=44–49%, Odontostyle=40–45 μm; ♂ L=1.31–1.53 mm, a=22.5–28, b=4.3–5.1, c=49–71.5, c’=0.5–0.9, Odontostyle=39–44 μm, Spicules=49–61 μm) (Iran)	*Enchodelus babakicus* (Pedram et al., 2009)
4	Uterus short (1–2 times corresponding body diam.) (♀ L=1.38–1.92 mm, a=19–32, b=4.0–6.2, c=55–91, c’=0.5–0.7, V=37–47%, Odontostyle=37–44 μm; ♂L=0.94–2.16 mm, a=19–39, b=3.6–6.0, c=41–100, c’=0.6–0.9, Odontostyle=24–33 µm, Spicules=46–70 µm) (Holarctic region)	*Enchodelus macrodorus* (de Man, 1880) Thorne, 1939)
–	Uterus long(> 2 times corresponding body diam.)	5
5	Body length <1.3 mm; (♀ L=0.94–1.29 mm, a=19–28, b=3.5–5.6, c=47–73, c’=0.5–1.0, V=43–55%, Odontostyle=37–45 μm; ♂ L=1.24–1.28 mm, a=26–37, b=4.6–4.8, c=52–54, c’=0.7–0.8, Odontostyle=38–40 μm, Spicules = 45–50 μm) (India, Korea)	*Enchodelus microdoroides* (Baqri & Jairajpuri, 1974)
–	Body length >1.5 mm	6
6	Dorsal cell mass near cardia present. (♀ L=1.59–1.87 mm, a=21.1–28.6, b=4.3–5.3, c=55.3–87.5, c’=0.5–0.7, V=41.7–49.7%, Odontostyle=39.5–47 μm) (Romania)	*Enchodelus carpaticus* (Ciobanu et al., 2010)
–	Dorsal cell mass near cardia absent	7
7	Lip region narrow < 18 µm, males present (♀ L=1.57–2 mm, a=21.6–33.1, b=4.4–5.7, c=45.8–70.3, c’=0.6–0.9, V=45–51%, Odontostyle=38–43 µm; ♂ L=1.49–1.79 mm, a=19.6–29.8, b=4.4–5.4, c=46.4–58.9, c’=0.7–0.8, Odontostyle=39–44.5 µm, Spicules=65–74 μm) (Russia – Severnaya Zemlya Archipelago)	*Enchodelus makarovae* sp. n.
–	Lip region wide, > 19 μm, males absent (♀ L=1.54–2.5 mm, a=20.3–25.9, b=4.0–6.0, c=40–104, c’=0.4–0.8, V=40–49%, Odontostyle=43–51 μm) (Greenland, Spain, Albania, Iran, Russia – Putorana Plateau)	*Enchodelus groenlandicus* (Ditlevsen, 1927)


## Supplementary Material

XML Treatment for Enchodelus makarovae

XML Treatment for Enchodelus groenlandicus
